# Deep learning-based corrosion inspection of long-span bridges with BIM integration

**DOI:** 10.1016/j.heliyon.2024.e35308

**Published:** 2024-07-30

**Authors:** Kotaro Hattori, Keiichi Oki, Aya Sugita, Takeshi Sugiyama, Pang-jo Chun

**Affiliations:** a IHI Infrastructure Systems Co., Ltd, 3 Ohama Nishimachi, Sakai, Osaka, 590-0977, Japan; b JB Toll Systems Co., Ltd, 3-2-17 Isobe-dori, Chuo-ku, Kobe, Hyogo Prefecture, 651-0084, Japan; c Department of Civil Engineering, The University of Tokyo, 7-3-1 Hongo, Bunkyo-ku, Tokyo, 113-8656, Japan

**Keywords:** *Corrosion*, *Long-span bridge*, *Deep learning*, *BIM*, *Optical flow*

## Abstract

Infrastructure operation and maintenance is essential for societal safety, particularly in Japan where the aging of infrastructures built during the period of high economic growth is advancing. However, there are issues such as a shortage of engineers and inefficiencies in work, requiring improvements in efficiency and automation for their resolution. Nevertheless, there are still many inefficiencies in the current procedures for bridge inspections. Usually, inspection engineers check for damage on bridges through close visual inspections at the site, then photograph the damaged parts, measure the size by touch, and create a report. A three-dimensional representation, considering the front and back of the structural elements, is needed for identifying damage, necessitating the creation of multi-directional three-dimensional drawings. However, this process is labor-intensive and prone to errors. Furthermore, due to the lack of uniformity in records, it is challenging to refer to past inspection histories. Especially for long bridges, without resolving such issues, the required labor and the number of mistakes could exceed acceptable limits, making proper management difficult. Therefore, in this study, we developed a method for automatically measuring the position and area of corroded parts by capturing images of the lower surface of the stiffening girder using a bridge inspection vehicle and utilizing image diagnosis technology. By integrating these results into a 3D model called BIM (Building Information Modeling), it becomes possible to manage the bridge more efficiently. We verified this method on actual long bridges and confirmed its effectiveness.

## 1 Introduction

Proper operation and maintenance of infrastructure are crucial for ensuring people's safety. Particularly in Japan, many infrastructures built during the period of high economic growth are aging, making immediate action a pressing issue. Therefore, inspections are required to accurately assess the condition of infrastructure structures. However, there is often a shortage of inspection technicians relative to the number of infrastructure structures, and the need for repair work due to aging of infrastructure further accelerates this shortage of manpower. Therefore, there is a strong demand for efficiency and automation in operations. Additionally, in several countries including Japan, the declining birthrate and aging population have exacerbated the shortage of skilled technicians and the lack of young technicians. Moreover, the construction industry has longer average working hours compared to other industries, resulting in the problem of young technicians leaving the industry. Considering these factors, improving productivity is a crucial issue that must be addressed.

The method of bridge inspection varies depending on the country and the administrator. For example, in Japan, the name of the bridge, its location, structural division, component name, type of damage, location and quantity of damage, images of the damage, and health assessment are recorded in a format and are typically saved as a PDF. Structures are public structures, and while operation and maintenance are typically organized by public institutions, there are no dedicated managers, necessitating the creation of a format for accurate information succession. The creation of this format is performed as an indoor task after the inspection worker confirms the presence or absence of bridge damage on-site through close visual inspection, then photographs the damaged part and measures its size by touch. However, this method has the following inefficiencies.

For example, a large number of photos are taken during bridge inspections, and it is often the case that it becomes unclear which part of the damage the photos are depicting, necessitating a revisit to the site. Additionally, the format is often unstructured and inconsistent, making it difficult to refer to or integrate past inspection histories and repair records. Furthermore, when based on a PDF, the location of damage is indicated using 2D floor plans and detail drawings. However, considering the front and back surfaces of the components, it is necessary to accurately indicate the three-dimensional position of the damage. Therefore, in the case of a three-dimensional structure like a truss, it becomes necessary to create drawings viewed from multiple directions such as side, plan, and cross-section. Consequently, the burden of creating drawings on inspection technicians is significant, and in addition, sharing information about the location of damage with third parties other than the inspection technicians takes time. Especially for long-span bridges, if such issues are not resolved, the required labor and the number of mistakes could exceed acceptable limits, making proper management difficult.

These results suggest that the current operation and maintenance, such as inspections and repairs, are inefficient as they rely on manual work by engineers to ensure accurate information succession. Therefore, this paper proposes a new management method using BIM (Building Information Modeling). BIM is a method to improve the productivity of operation and maintenance by associating attribute information such as bridge name, structural classification, and member name with a 3D model and managing the position of damage within the 3D model. The history of inspections and repair work is managed using BIM, which allows for systematic referencing and integration of data. As a result, it becomes possible to structure data through automated input of attribute information, and to link damage photos with the 3D model, thereby realizing advanced and labor-saving operation and maintenance.

While a three-dimensional BIM can hold and utilize a significantly larger amount of information in a structured manner compared to a two-dimensional model, obtaining damage data to input into it is not necessarily straightforward. Rather, by strictly organizing the data schema, vague methods of obtaining damage information - as to both location and degree - that were operational in non-unified and unstructured formats as before, would undermine the benefits of the BIM.

In this study, we propose a method for the automated detection of damage on the underside of steel box girder bridges, starting from the preliminary stage of photography. To date, many studies have been conducted on the automatic detection of infrastructure damage using deep learning. Particularly, there has been extensive research on concrete cracking, including studies using Mask R–CNN and a custom-developed semantic segmentation model by the authors et al. [[1], [2], [3], [4], [5]], a study using CNN by Cao et al. [6], and research using U-Net by Zhenqing and Lingxin et al. [7,8]. On the other hand, there are not many studies on the detection of corrosion damage in steel materials. There are studies such as the one by Qinghua et al. [9], which detected steel bridge corrosion from UAV photography results, and a study by the authors et al. [10]. In this study, based on the perspective that the evaluation of the area of corrosion damage is extremely important for the efficient calculation of the quantity of repair work, we construct a damage detection method based on PointRend [11] with the aim of maximizing accuracy.

In addition, this study also establishes a method for capturing images. While there are many studies that use UAVs to photograph bridges and detect damage, long-span bridges, which are the target of the method developed in this study, are often located over the sea, and in Japan, it is often difficult to take pictures with UAVs due to flight restrictions. On the other hand, for such long-span bridges, inspection vehicles are often installed for access to hard-to-reach members. Therefore, this study also proposes a new method of obtaining images of the outer surface of the girder using inspection vehicles. Furthermore, we conducted a demonstration experiment on a long-span bridge connecting Honshu and Shikoku in Japan and discussed its effectiveness.

## 2 Method

**Fig. 1:**
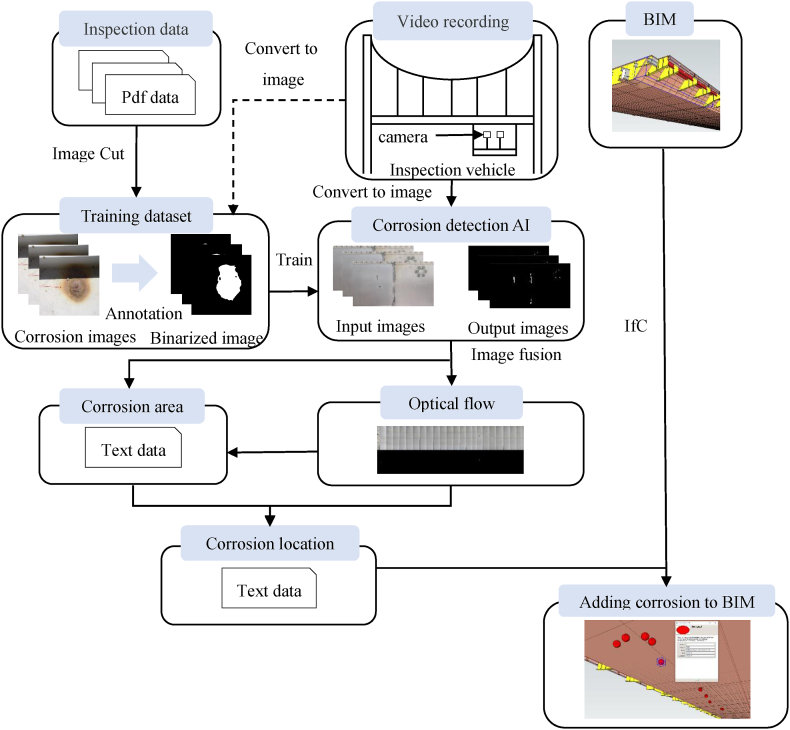
Inspection flow.

Fig. 1 shows the inspection flow proposed in this study. We use part of the images from past inspection data and those taken during the demonstration experiment to perform machine learning and accuracy evaluation. The captured images are used to detect corrosion through image diagnosis, and the area of the corrosion is calculated. Image fusion is performed using the captured images and the image diagnosis images to identify the position of the corrosion. The position and area of the corrosion are stored as text data, and the corrosion data is managed using BIM.

### 2.1 Semantic segmentation

#### 2.1.1 Overview

In this study, we construct a method for automatic corrosion damage detection using Semantic segmentation. Semantic segmentation is a technique to extract object regions at a pixel level from an image, and due to recent advancements in deep learning technology, it is now possible to extract regions with high accuracy. One of the early semantic segmentation methods using deep learning is the Fully Convolutional Network (FCN) [12], based on the image analysis method, Convolutional Neural Network (CNN). CNN is generally used as a classification model, in which case a fully connected layer is provided in the final layer. However, in semantic segmentation, the feature map extracted from the input image by convolution is output without using a fully connected layer in the final layer. This feature map becomes an image that indicates the probability of classification classes at each pixel. Furthermore, FCN integrates the feature maps output in the intermediate layers at the final layer, and it can output not only the feature map of the final layer but also more detailed region extraction results. As a more accurate segmentation method, Mask R–CNN [13], based on Faster R–CNN [14], has been proposed. Faster R–CNN detects object regions from the input image using CNN and classifies those objects. While it is generally possible to detect corrosion using such an object detection algorithm, pixel-level segmentation along the shape of corrosion is necessary to accurately identify the area of corrosion and evaluate its size. Mask R–CNN achieves high-accuracy segmentation by extracting regions from parts of the object regions detected by Faster R–CNN. In this study, we perform extraction of corrosion areas based on Mask R–CNN. However, segmentation methods using CNN tend to have low extraction accuracy at the boundary between objects and the background. This is because when classifying each pixel of an image, calculations are performed evenly for all pixels, and unnecessary calculations are performed in low-frequency areas other than the boundary between objects and the background. Therefore, to reduce computation costs, the image is divided into a low-resolution grid and predictions are made. As a method to improve the extraction accuracy at the boundary between objects and the background while suppressing computation costs, PointRend has been proposed.

**Fig. 2:**
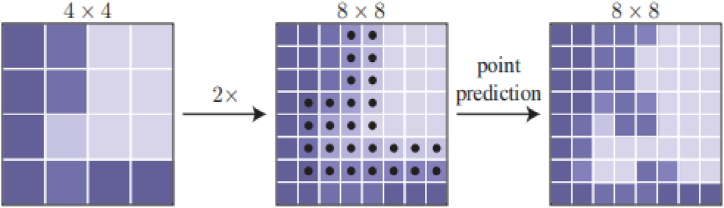
Example of one adaptive subdivision step.

PointRend repeatedly renders bilinear interpolation on a low-resolution coarse grid to upsample the labels of the segmentation predicted on the previous grid, as shown in Fig. 2 [9], with greater accuracy. In this upsampling, *N* uncertain points are chosen (for example, points where the probability of a binary mask is closest to 0.5). In PointRend, features are calculated for each of these *N* points, and labels are predicted. If the resolution of the input is $M_{0}\times M_{0}$ and the resolution of the output is M×M, then PointRend requires $N\;\log_{2}\left(M/M_{0}\right)$ predictions. This means that it can get by with fewer estimations compared to the usual M×M segmentation prediction. Therefore, it enables efficient inference.

**Fig. 3:**
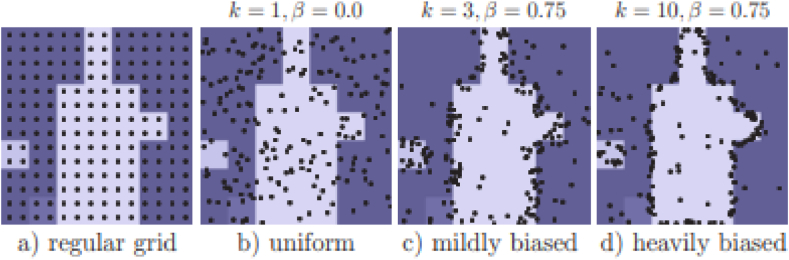
Point sampling during training.

In the training of PointRend, point selection becomes a crucial element. This selection is based on the following three principles and is designed to bias towards uncertain areas while maintaining a certain degree of uniform coverage. This allows PointRend to achieve efficient learning:

1. Over generation: we over-generate candidate points by randomly sampling kN points (k>1) from a uniform distribution.
2. Importance sampling: we focus on points with uncertain coarse predictions by interpolating the coarse prediction values at all kN points and computing a task-specific uncertainty estimate. The most uncertain βN points (β ∈ [0, 1]) are selected from the kN candidates.
3. Coverage: the remaining (1−β)N points are sampled from a uniform distribution. We illustrate this procedure with different settings, and compare it to regular grid selection, in Fig. 3 [9].

During training, the prediction and loss function are calculated only for the *N* sample points, in addition to the coarse segmentation. This is simpler and more efficient than backpropagation through subdivision steps.

**Fig. 4:**
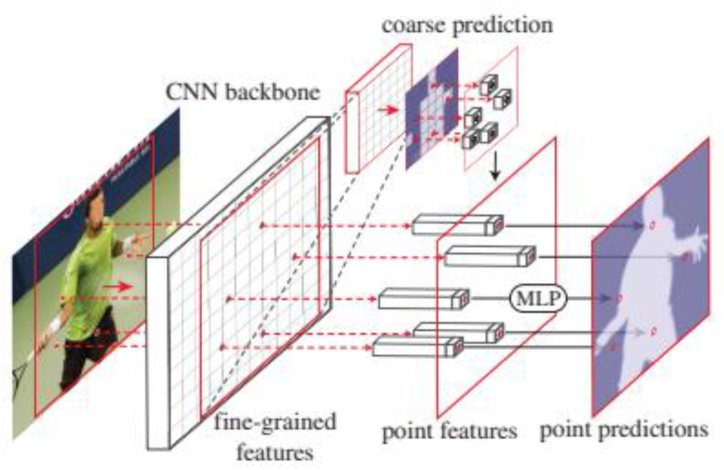
PointRend applied to instance segmentation.

PointRend forms the Point Feature by combining the Coarse Prediction Features and Fine-Grained Features, as shown in Fig. 4 [9].

PointRend can be flexibly integrated into existing segmentation models, thus in this study, we construct a corrosion area extraction algorithm using Mask R–CNN integrated with PointRend.

#### 2.1.2 Training of the machine learning model

**Fig. 5:**
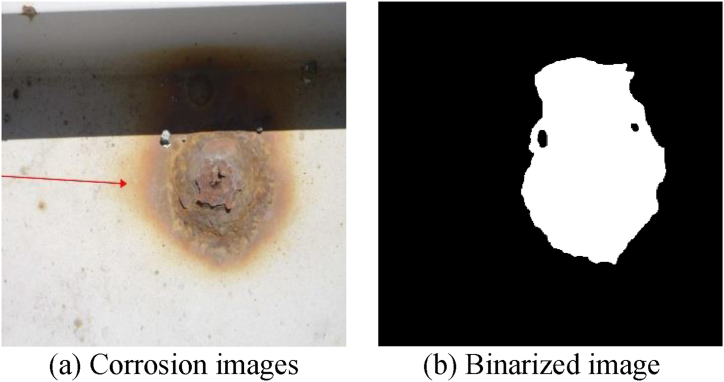
Input/output data.

In this study, we perform semantic segmentation, a type of supervised machine learning, to determine whether specific pixels in an image are corroded or not. In supervised machine learning, humans provide input and output data prepared in advance as training data, and the machine learns the relationship between input and output, and can predict the output for unknown input data. In this study, we construct a decision-making algorithm by preparing corroded images for input in advance and creating binary images (Fig. 5) that distinguish between corroded areas and other areas for learning. This algorithm allows us to determine the range of corrosion at the pixel level, even in unknown corroded images that have not been used for training.

**Fig. 6:**
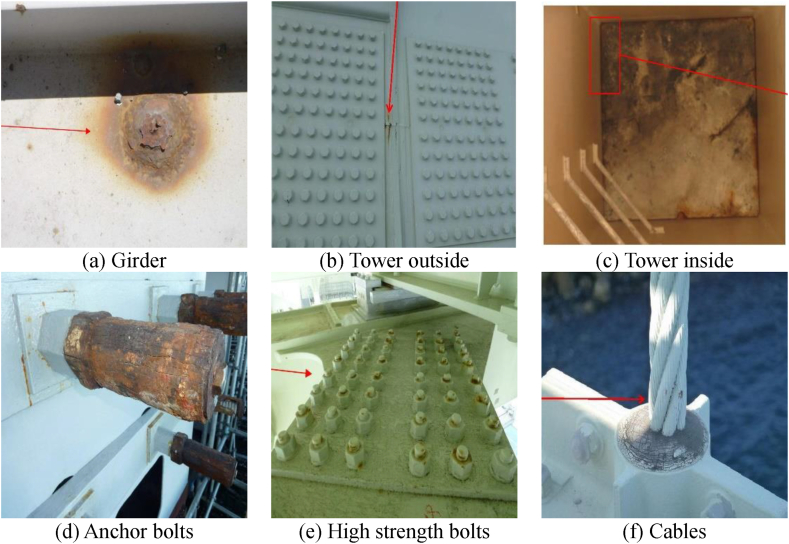
Examples of corrosion images for each item.

The input and output data used are the long-span bridge group inspection data from 2014 to 2022, including the Akashi Kaikyo Bridge, owned by Honshu-Shikoku Bridge Expressway Co., Ltd. After trimming the images of the inspection reports that were converted into PDF, corrosion areas were annotated by professional engineers specializing in steel bridges. The image sizes range widely from 333 × 262px to 4676 × 3307px.The details of the corroded images used are shown in Table 1. Examples of corroded images for each item are shown in Fig. 6. The red frames and arrows in the images indicate the position of corrosion. In past inspection records, arrows were directly written on the images, making partial deletion difficult, so they were used as is for input and output data.

**Table 1:** Number of photos by item.

Item	Number of photos
Steel material	1813
Bolts	2079
Cables	8
Total	3900

**Fig. 7:**
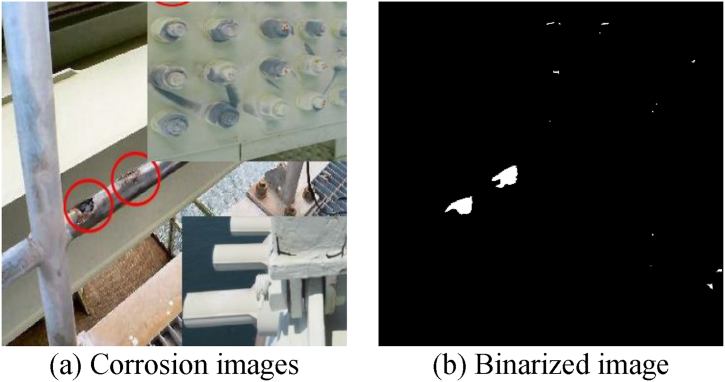
Example of training data using CutMix.

As shown in Fig. 6, the distance to the subject in corrosion photographs of long-span bridges is not constant. Some images can only be taken from a distance, as in the case of main towers, while others can be taken at close range using inspection vehicles. Furthermore, the shape of corrosion varies, such as linear corrosion that occurs on cables, and circular corrosion that occurs on flat shapes like girders. In other words, a different approach is needed from the techniques used to detect cracks in concrete [[1], [2], [3], [4], [5]]. Additionally, there is a level of difficulty when compared to the detection of concrete cracks, which are predominantly linear. Therefore, considering the various patterns of corrosion images, it was considered difficult to improve the accuracy with only the images shown in Table 1, as the training data was insufficient. In such cases, increasing the training data by data augmentation is a solution, but it was judged that general data augmentation techniques such as image rotation and inversion do not cover the diversity of corrosion damage. Therefore, in this study, data augmentation was performed using a technique improved from CutMix [15], which was developed by the authors el at [16]. CutMix is a technique that partially extracts and combines the features of Cutout [17] and Mixup [18]. The training data is augmented by the CutMix method, which creates new images by connecting multiple images with labels, as shown in Fig. 7. By connecting the labels of the training data, a decrease in learning efficiency can be prevented, and higher accuracy can be achieved compared to similar methods such as Cutout and Mixup. The reason for using this method is that corrosion damage often presents cases where the shapes are similar but the sizes differ. With this in mind, it is considered that randomly enlarging and reducing during the CutMix process does not have a negative effect. Furthermore, since changes in features due to rotation direction often do not occur, rotating is also natural and can lead to improved accuracy.

**Table 2:** Accuracy of detection results.

	Case1	Case2
Training data	3120	24448
Validation data	390	3056
Test data	390	390
Total	3900	27894

In this study, accuracy verification was conducted using the images shown in Table 1. Table 2 shows the breakdown of training data, validation data, and test data. In Case 1, the original images from Table 1 were used, with 80 % as training data, 10 % as validation data, and 10 % as test data. In Case 2, data augmentation was performed on the training data of Case 1 using CutMix, and the dataset was constructed so that the ratio of training data to validation data was 8:1. These were resized to 1024 × 1024px and used as training data. The Mask-R-CNN model used in this study used Resnet50 [19] as the backbone, and the weights trained using the MSCOCO [20] dataset were used as the initial weights for fine-tuning the model. During training, the analysis was conducted with a batch size of 4 per step.

#### 2.1.3 Accuracy verification

**Fig. 8:**
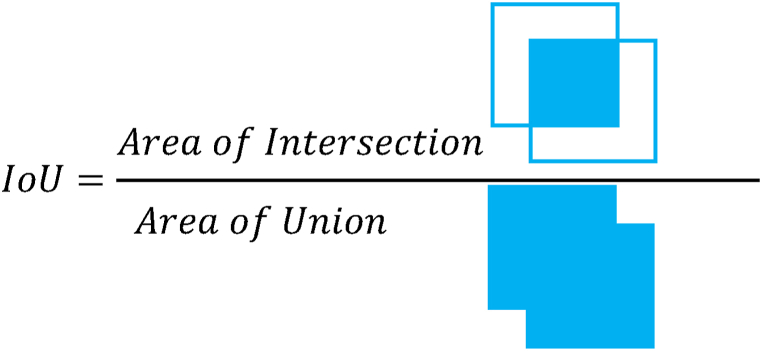
*IoU*(Intersection over union).

In this section, we used the trained model to detect corrosion parts from the test data and performed accuracy evaluation. The evaluation used a confusion matrix, which is commonly used in machine learning. In Table 3, TP represents True Positive, FN represents False Positive, TN represents True Negative, and FP represents False Positive. From the values of these confusion matrices, six values (accuracy, precision, recall, specificity, F1 score *IoU*) were calculated and evaluated. These indicators are defined by Eqs. (1), (2), (3), (4), (5), (6). The *IoU* in Eq. (6) is an indicator that shows how much the two regions overlap, which is derived from Fig. 8.

(1) $$Accuracy=\frac{TP+TN}{TP+FN+FP+TN}$$

(2) $$Precision=\frac{TP}{TP+FP}$$

(3) $$Recall=\frac{TP}{TP+FN}$$

(4) $$Specificity=\frac{TN}{FP+TN}$$

(5) $$F1\;score=\frac{2\times \left(Precision\times Recall\right)}{Precision+Recall}$$

(6) $$IoU=\frac{TP}{TP+FP+FN}$$

$$IoU=\frac{Area\;of\;Intersection\;}{Area\;of\;Union\;}$$

**Table 3:** Confusion matrix for accuracy evaluation.

Prediction Result	Corrosion	No Corrosion
Corrosion	TP	FP
No Corrosion	FN	TN

Using Eqs. (1), (2), (3), (4), (5), (6), the results of Case 1 and Case 2 described in 2.1.2 are shown in Table 4. The evaluation values were higher for the CutMix data, excluding recall.

**Table 4:** Accuracy of detection results.

	Case1	Case2
Accuracy rate	0.945	0.962
Precision rate	0.408	0.515
Recall	0.833	0.808
Specificity	0.950	0.968
F1 Score	0.548	0.629
IoU	0.377	0.459

**Fig. 9:**
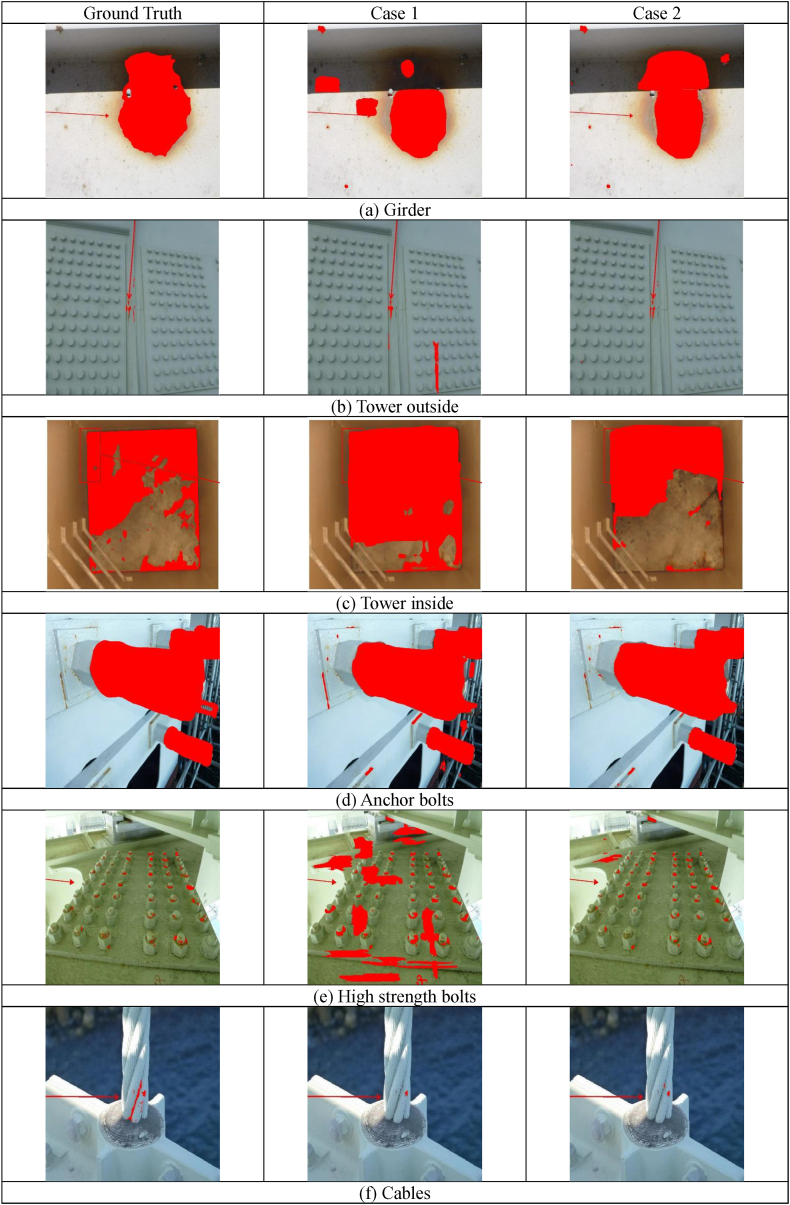
Example of detection result.

The results of the accuracy evaluation of the corrosion image shown in Fig. 6 are illustrated in Fig. 9. The corroded areas are marked in red, and from left to right, the ground truth, Case 1, and Case 2 are displayed. The girder of (a), Case 1 is not able to detect the corrosion in the shadowed areas, while Case 2 can. This shows a trend close to the ground truth. In the outer surface of the main tower in (b) and the anchor bolts in (d), both cases can detect corrosion, but Case 1 detects more non-corroded areas compared to Case 2. In the inner surface of the main tower in (c), both cases detect corrosion, but the corrosion area range in Case 2 is closer to the ground truth. In the high-strength bolts in (e), only the head of the bolt should be detected as corroded, but Case 1 has many false detections. Case 2 has some false detections, but it generally matches the correct data. In the cables in (f), corrosion is distributed along the parts where the strands come into contact, but neither Case 1 nor Case 2 can detect it. This is believed to be due to the small number of cable images shown in Table 1. Even when expanding the training data with CutMix, it was still considered insufficient as training data. It is not a problem for the steel box girder bridges that are the target this time, but when the target structure contains many cables, it is necessary to increase the sample of corrosion images or increase the training data through data augmentation.

From the results shown in Table 4 and Fig. 9, it can be said that data augmentation using CutMix is effective for this model.

### 2.2 Optical flow

In this section, we propose improvements to the optical flow method for automatically transcribing to BIM by identifying the location, as it is inefficient to directly transcribe widespread corrosion to BIM for the purpose of labor-saving.

Optical flow refers to the optical movement of each point between two temporally continuous image frames. It is observed by projecting the movements of objects, surfaces, edges, etc. that visually occur between the observer and the observed scene onto an image. In image estimation using optical flow, it is assumed that the brightness of objects within an image does not significantly change between continuous frames, and that adjacent pixels move similarly. Here, we define a pixel in the first image as I(x,y,t). It is assumed that this pixel moves a distance of (dx,dy) in the second image taken at a time dt later. These two pixels are considered identical, establishing the following relationship.

(7) I(x,y,t)=I(x+dx,y+dy,t+dt)

By expanding the right side of Eq. (7) using Taylor's series, removing common terms, and then dividing by dt, we obtain the following equation:

(8) $$I_{x}v_{x}+I_{y}v_{y}+I_{t}=0$$

We define the vector $V=\left(v_{x},v_{y}\right)$ as the velocity vectors in the x and y directions, respectively. Both $v_{x}$ and $v_{y}$ are variables, and the solution cannot be obtained from Eq. (8) alone. In this study, we use the Lucas-Kanade method [21] to find the solution.

The Lucas-Kanade method assumes that pixels in a local neighborhood within an image have the same velocity, and it solves the optical flow equation for all pixels in that neighborhood using the least squares method [22]. If the number of pixels of interest is denoted as n, Eq. (9) is obtained.

(9) $$\begin{array}{c} I_{x}\left(\;x_{1},y_{1}\right)∙v_{x}+I_{y}\left(\;x_{1},y_{1}\right)∙v_{y}=-I_{t}\left(\;x_{1},y_{1}\right)\\ I_{x}\left(\;x_{2},y_{2}\right)∙v_{x}+I_{y}\left(\;x_{2},y_{2}\right)∙v_{y}=-I_{t}\left(\;x_{2},y_{2}\right)\\ \vdots \\ I_{x}\left(\;x_{n},y_{n}\right)∙v_{x}+I_{y}\left(\;x_{n},y_{n}\right)∙v_{y}=-I_{t}\left(\;x_{n},y_{n}\right) \end{array}$$

Using matrix vector notation, Eq. (9) can be rewritten as Eq. (10).

(10) $$\underset{A}{\underbrace{\left(\begin{array}{cc} I_{x}\left(\;x_{1},y_{1}\right) & \;I_{y}\left(\;x_{1},y_{1}\right)\\ I_{x}\left(\;x_{2},y_{2}\right) & I_{y}\left(\;x_{2},y_{2}\right)\\ \vdots & \\ I_{x}\left(\;x_{n},y_{n}\right) & I_{y}\left(\;x_{n},y_{n}\right) \end{array}\right)}}∙\underset{v}{\underbrace{\left(\begin{array}{c} v_{x}\\ v_{y} \end{array}\right)}}=-\underset{b}{\underbrace{\left(\begin{array}{c} I_{t}\left(\;x_{1},y_{1}\right)\\ I_{t}\left(\;x_{2},y_{2}\right)\\ \vdots \\ I_{t}\left(\;x_{n},y_{n}\right) \end{array}\right)}}$$

This system typically has more equations than unknowns, resulting in an overdetermined system. The Lucas-Kanade method seeks a compromise solution using the least squares method. As a result, a 2 × 2 system is solved.

(11) $$\begin{array}{c} A^{T}Av=A^{T}b\\ or\\ v={\left(A^{T}A\right)}^{-1}A^{T}b \end{array}$$

in this study, we filmed a steel bridge and converted the video into still images. While optical flow generally tracks the movement of objects, in this study, we used it in reverse to extract the movement of the camera, which is the viewpoint, from a stationary point. We examined whether we could map the corroded areas by merging these images using optical flow.

### 2.3 Corrosion area, amount and location of corrosion

**Fig. 10:**
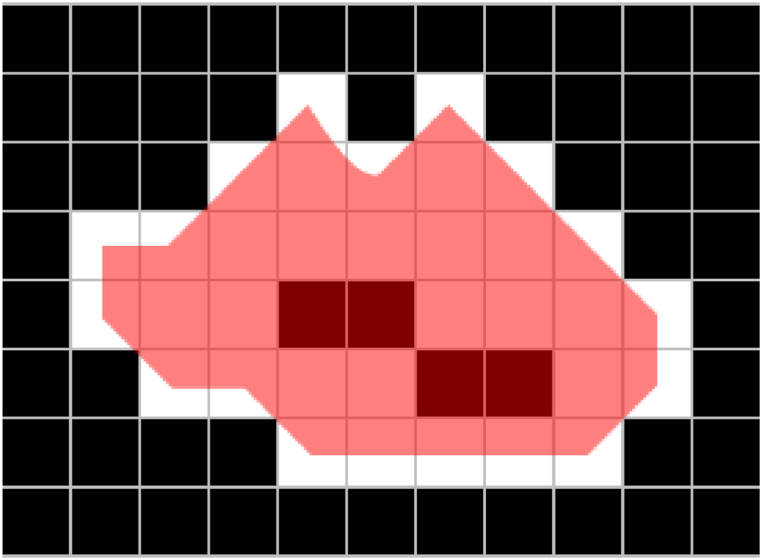
Corrosion area.

The corroded area is calculated using contour tracking. The corroded area can be calculated by subtracting the black pixels, i.e., the healthy parts, within the red area in Fig. 10. However, if the area of the healthy parts is small, considering that the corroded and healthy. parts are repaired together with a brush, the red area is recorded as the corroded area. However, if the area of the healthy parts within the red area is large, contour tracking is applied to the black parts, and they are excluded from the corroded area.

The corroded area calculated by contour tracking is limited to cases where pixels are adjacent to each other. Therefore, scattered corrosion is accounted for individually. When actually recording the quantity of corrosion, scattered corrosion is grouped and counted as one corrosion for data management purposes. This grouping counts those with a gap of 50 mm or less between the corroded areas as one group, and dilation processing is used for the calculation. The conversion between pixels and length units will be described in Chapter 2.4.

The position of the corrosion is calculated using the centroid of the grouped corroded area. Since the centroid is output in pixel units, it is converted to length units. The centroid is used to represent the position of corrosion reflected in BIM.

### 2.4 How to reflect in BIM

The information on the location and area of the corroded parts presented in chapters 2.1 to 2.3 is in pixel units, and needs to be converted to length units to be reflected in the BIM. To convert to length units, the width *W* and height *H* of the subject are calculated from the camera's focal length *f*, the distance *L* from the lens to the subject, and the width *w* and height *h* of the image sensor.

(12) $$W=wｘ\frac{L}{ｆ},H=hｘ\frac{L}{ｆ}$$

Eq. (12) is valid only when the subject and the lens are facing each other directly, and cannot be calculated with this equation if they are not. The details of the shooting method will be described in Chapter 3, but in this paper, we will proceed on the assumption that the subject and the lens are facing each other directly.

The corrosion center (x,y) is calculated using the center point $P=\left(P_{x},P_{y}\right)$ of the corrosion area on the image and *L*, *f* from Eq. (12), converted into units of length.

(13) $$\left(x,y\right)=\frac{L}{f}\left(P_{x},P_{y}\right)$$

To reflect the center of the corrosion area calculated from image diagnosis into BIM, a coordinate transformation is necessary. The coordinates (X,Y) of BIM are calculated using the following formula with a rotation matrix and parallel translation.

(14) $$\left(\begin{array}{c} X\\ Y \end{array}\right)=\left(\begin{array}{cc} \cos\;\theta & \sin\;\theta \\ -\sin\;\theta & \cos\;\theta \end{array}\right)\left(\begin{array}{c} x\\ -y \end{array}\right)+\left(\begin{array}{c} a_{x}\\ a_{y} \end{array}\right)$$

θ is the rotation angle, and $a=\left(a_{x},a_{y}\right)$ indicates the rotation origin.

**Fig. 11:**
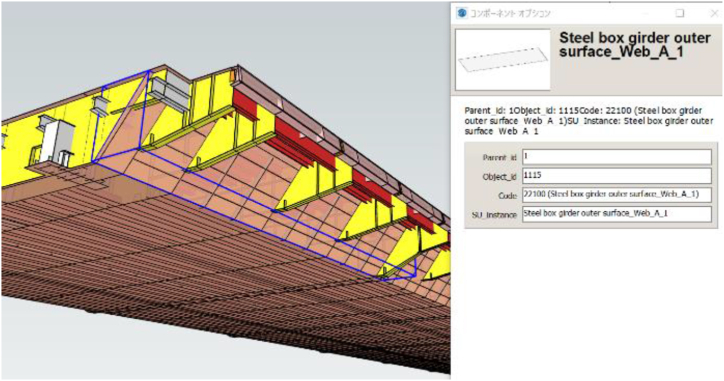
Example of BIM

As mentioned in Chapter 1, BIM includes the structural division and member names, so the structural member can be identified just by passing the position information from Eqs. (13), (14). An example of BIM is shown in Fig. 11.

## 3 Target bridges and inspection methods

### 3.1 Target bridges

**Fig. 12:**
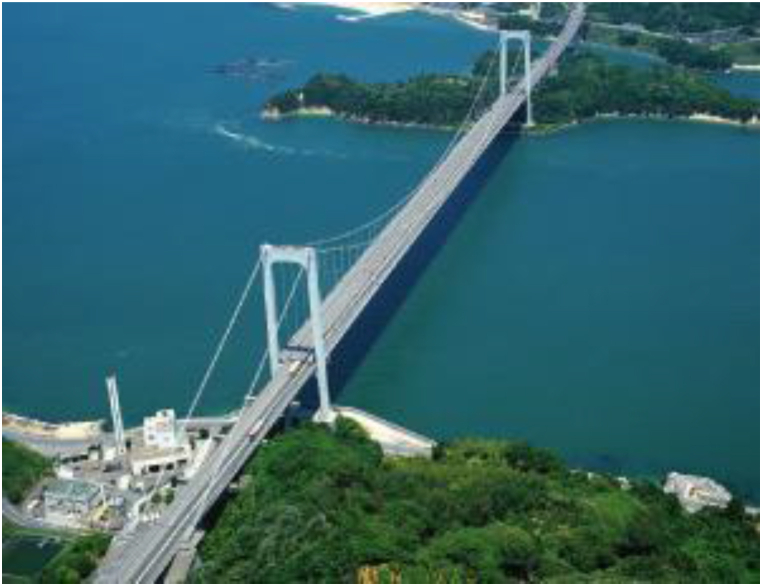
Oshima bridge.

In this paper, we conduct an experiment of the method developed at Oshima Bridge (Fig. 12), managed by Honshu-Shikoku Bridge Expressway Co., Ltd. Oshima Bridge is one of the long-span bridges situated over the sea, connecting Honshu, the largest island in Japan, and Shikoku, the fourth largest island in Japan, via an automobile-only road. It is the first suspension bridge in Japan to adopt a steel box girder as a stiffening girder. The structural details and average traffic volume are shown in Table 5.

**Table 5:** Structural specifications.

Structural form	Single span stiffened box girder suspension bridge
Center span length	556.2m
Width	23.7m
Lower flange width	17.0m
Average traffic volume	9656 vehicles/day(2022)

Most of the long-span bridges in the strait are made of steel, and it is crucial to control corrosion to maintain their function over the long term. The most fundamental maintenance method to achieve this is painting.

Oshima Bridge was completed in 1987, and 35 years have passed since its construction. The coating of Ohashi Bridge is a heavy-duty anti-corrosion coating that uses an inorganic zinc-rich paint with a high sacrificial anti-corrosion effect on the steel surface, and a polyurethane resin paint was used for the top coating at the time of construction. As the service life of polyurethane is about 20 years, there are already places where the intermediate coating has started to be exposed. It is desirable to repaint before the undercoat is exposed and deteriorates. When the highly sacrificial anti-corrosion surface of the inorganic zinc-rich paint becomes exposed or disappears, re-coating becomes difficult at the site. Therefore, it is important to detect paint film deterioration early and take measures.

Next, we will explain the current situation of inspections for long-span bridges. According to Japanese law, regular inspections are conducted once every five years. However, the on-site work hours required for the girder inspection, in the case of the Oshima Bridge, which has a relatively small box girder, amount to over 150 man-days. In the case of the Akashi Kaikyo Bridge, it amounts to over 1000 man-days. Thus, the inspection process requires substantial costs.

An inspection vehicle, available for constant use, is installed in the stiffening girder section of the long-span.

Inspections using the inspection vehicle require a person to operate the inspection vehicle and another to inspect while observing the actual object, and multiple people are inspecting the exterior of the girder.

### 3.2 Shooting method and calibration

In this section, we propose a method for taking pictures of the underside of the stiffening girder of box girder using an inspection vehicle.

**Fig. 13:**
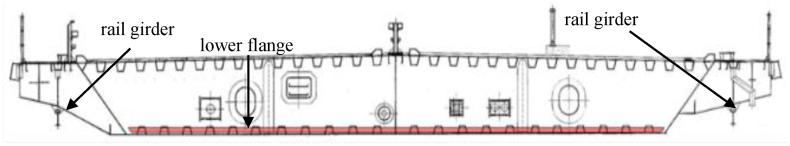
Section of stiffening girder rail girder.

The inspection vehicle is installed at a certain height from the underside of the stiffening girder, considering inspection and repair, and moves along the bridge axis on the rail girder under the bracket as shown in Fig. 13.

**Fig. 14:**
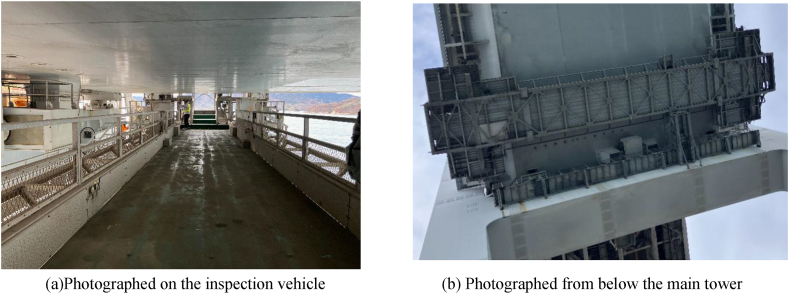
Inspection vehicle.

**Fig. 15:**
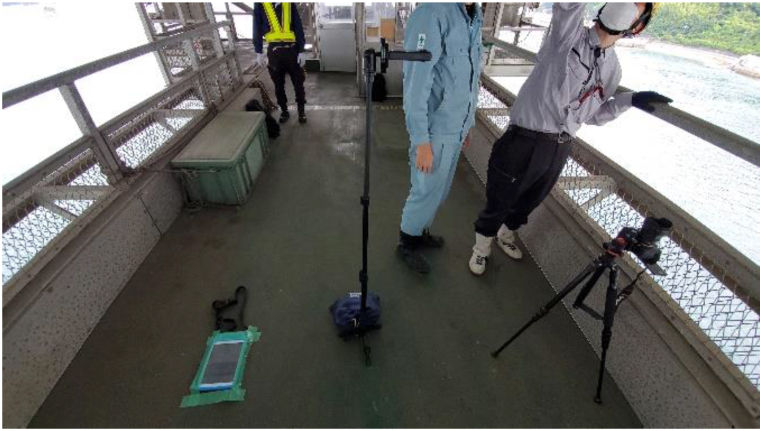
Camera installation.

**Fig. 16:**
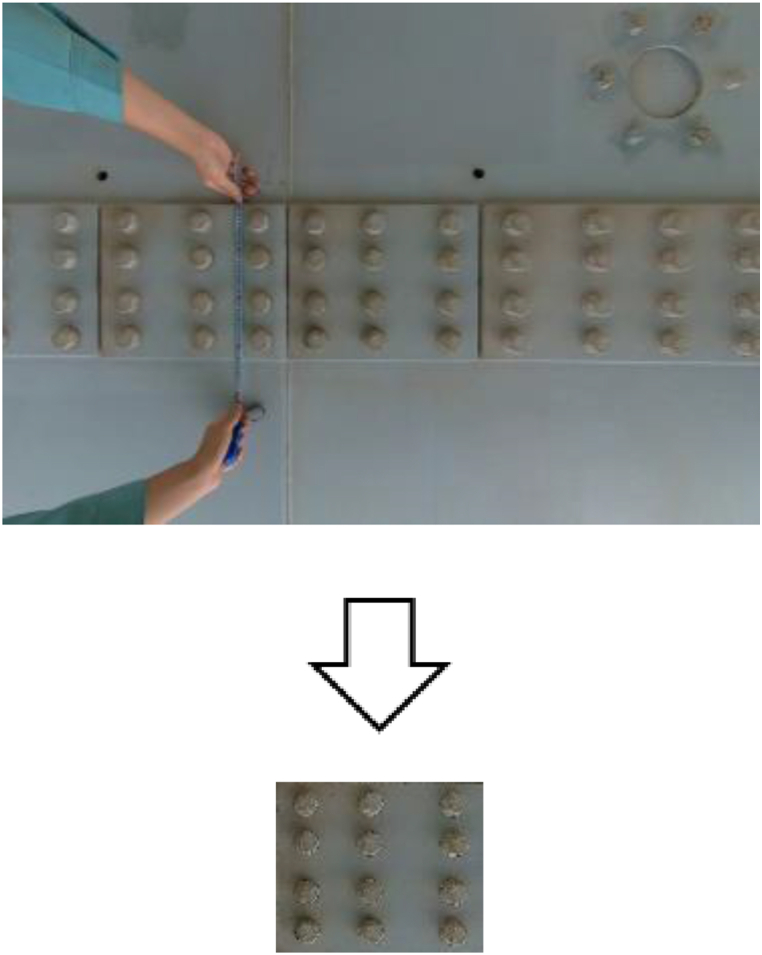
Measuring the splice plate (insta360).

As shown in Fig. 14, different types of cameras (iPad, digital camera, and Insta360) were arranged in the bridge axis direction on the inspection vehicle, and the underside of the stiffening girder shown in Fig. 13 was photographed. The specifications of each camera are shown in Table 6. The shooting range in the direction perpendicular to the bridge axis is about 2m for each camera. Since the lower flange width is 17m, it was necessary to set the shooting range. Therefore, the field-welded joint of the vertical seam of the lower flange, where a lot of corrosion is observed, was selected as the shooting range for this study. To take clear images with the inspection vehicle, as shown in Fig. 15, the iPad was placed directly on the road surface of the inspection vehicle, fixed with tape, and shot with an in-camera. The digital camera and Insta360 were fixed using a tripod. By moving the inspection vehicle in the bridge axis direction and shooting video with the camera, efficient inspection was made possible. The camera angle was set to face the subject directly, and the shooting magnification was fixed. Also, by keeping the distance from the camera to the subject constant, the value of *L/f* in Eq. (12) was set to be constant while shooting. The method of calculating *L/f* was to calculate the dimensions of the subject with clear dimensions and the image size when extracting a still image from the video, calculate *W/w* or *H/h*, and calculate *L/f* in Eq. (12). An example of the target image and the calculated results are shown in Fig. 16 and Table 7, respectively.

**Table 6:** Camera specifications.

	iPad	digital camera	Insta360
Model	iPad (7th generation)	α7 IV	Insta360 ONE X2
Manufacturer	NTT DOCOMO, INC.	SONY CORPORATION	Shenzhen Dvision Co., Ltd.

**Table 7:** Measurement result (iPad).

*H*(mm)	320
*H*(px)	177
*H/h* = *L/f*(mm/px)	1.808

By utilizing such a photographic method, the influence of distortion in the central part could be minimized. With less distortion effect, it is believed possible to calculate the corrosion area from the captured images without using ortho-images.

### 3.3 Shooting and analysis results

#### 3.3. 1 Accuracy verification using CutMix

In chapter 2.1.3, we described how the utilization of a training data augmentation method, which improves CutMix, contributes to the enhancement of accuracy evaluation. Using the training data of CutMix, we conducted image diagnosis by converting the video shot in chapter 3.2 into still images. When we convert to still images at 1fps, there is a concern of generating a massive amount of data. However, if the overlap rate is set to 0 %, we cannot obtain an accurate corrosion area when there is corrosion at the edge of the image. Therefore, we extracted still images so that the overlap rate would be about 50 %. By setting it to 50 %, we can always shoot the same subject twice, and even if one is located at the edge of the image, the other can be positioned at the center of the image.

The image size in the axial direction of the bridge was calculated from Eq. (12), and the overlap rate was calculated by determining the movement amount of 1fps from the speed of the inspection vehicle. Assuming that the inspection vehicle runs at a constant maximum speed, we were able to secure an overlap rate of more than 50 %.

**Fig. 17:**
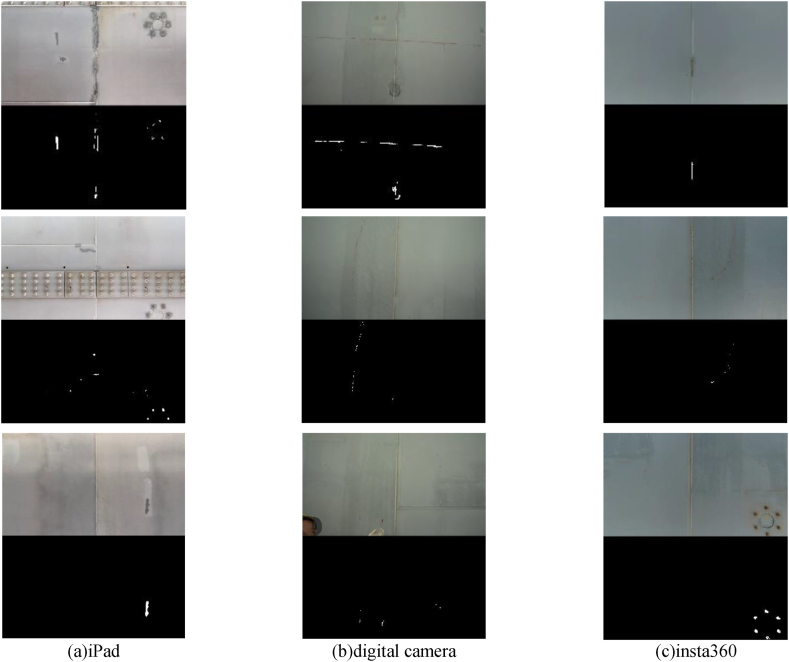
Corrosion diagnosis results.

The results of the image diagnosis constructed in Chapter 2 are shown in Fig. 17. From left to right, the results of the iPad, digital camera, and Insta360 are presented. The original image and the binarized image were arranged vertically to verify whether corrosion detection was possible. From the image results, it was confirmed from each image that corrosion, which should be observed with the naked eye, could be detected. This time, we took pictures with different cameras and verified the accuracy of corrosion detection, and it was confirmed from Fig. 17 that there was no problem with any camera.

#### 3.3. 2 Calculation of corrosion location and corrosion area

**Fig. 18:**
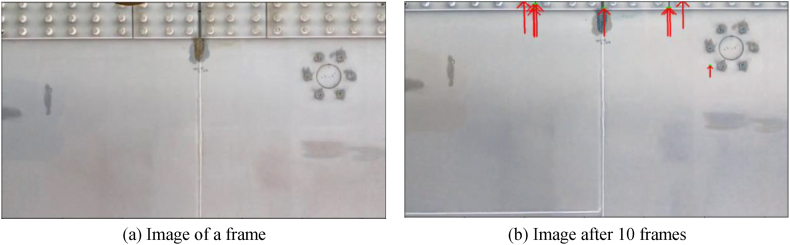
Feature point calculation using optical flow.

Still images were extracted from the video capturing the underside of the stiffening girder, and the vectors of feature points by optical flow are shown in Fig. 18 using an image of a certain frame and the image 10 frames later. In Fig. 18(b), the amount of movement (vector) of feature points from the image in (a) is shown. In this shooting, since the inspection vehicle is moving in the axial direction of the bridge, the vector is considered to be positive in the upward direction. However, there are also those not pointing upwards, suggesting the possibility of erroneous extraction of feature points. Therefore, only the upward vectors (85°–95°) were used this time, and the average amount of upward vectors was used as the movement amount to perform image fusion.

**Fig. 19:**
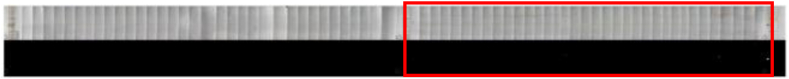
Image fusion using optical flow.

Fig. 19 shows the result of the iPad after the still images were cut out and fused by optical flow. The original image is placed on top and the binarized image is placed at the bottom. An image fusion was performed on the original image using optical flow. The original image and the binarized image are registered with the same file name, and image fusion is also performed on the binarized image with the same amount of movement as the original image. With image fusion, the image from the later frame is adopted for the overlapping range. When optical flow is implemented for each frame, a large amount of boundary lines for image fusion enters, making the corrosion part unclear, so a threshold for the frame to fuse images was set. This time, it was decided to fuse the next image when the image size in the axial direction of the bridge has moved more than 2/3.

**Fig. 20:**
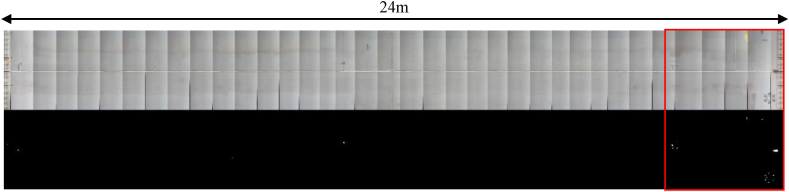
Image fusion result in block units.

Next, the method for calculating the corrosion area and the corrosion centroid is explained. In the image of Fig. 19, the joint part of the stiffening girder is captured, and the length between the joint parts is 24m from the drawing. The image between the joint parts in Fig. 19 is extracted and the image size is measured. The image enlarged by extracting the red-framed part of Fig. 19 is shown in Fig. 20.

**Fig. 21:**
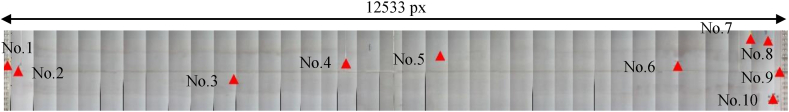
Consolidation of corroded parts by contour tracking processing and dilation processing.

For the binarized image in Fig. 20, contour tracking and dilation processes are performed to calculate the corrosion area and corrosion centroid. Fig. 21 shows the result of reflecting the corrosion centroid in the original image of Fig. 20. The red triangle marks in Fig. 21 indicate the centroid position, and each triangle mark is numbered. Table 8 shows the corrosion centroid and corrosion area corresponding to the numbers. For the corrosion area, the length was converted using *L/f* = 1.808 in Table 7.

**Table 8:** Corrosion location and corrosion area.

No.	Px(px)	Py(px)	Area(px)	Area(mm2)	x(mm)	y(mm)
1	55	554	38.5	126	105	1002
2	227	646	132.0	431	435	1168
3	3674	774	47.5	155	7036	1399
4	5469	523	282.5	923	10473	946
5	6978	399	7.5	25	13363	721
6	10778	563	696.5	2277	20640	1018
7	11943	133	170.5	557	22871	240
8	12221	160	188.0	615	23403	289
9	12406	657	2100.0	6865	23757	1188
10	12302	1091	1057.0	3455	23558	1973

The core of corrosion is converted to a unit of length using Eq. (13). In the bridge axial direction, using the joint length *W* = 24m and image size *w* = 12533px from Fig. 21, *W/w* = 1.915 is derived from Eq. (12). Furthermore, when Eq. (12) is rearranged, *W/w* becomes *L/f*. Here, *L/f* = 1.915 is substituted into Eq. (13) for calculation. In the bridge axial perpendicular direction, *L/f* = 1.808 from Table 7 is used and substituted into Eq. (13) for calculation. The results are shown in Table 8.

Here, the reason for changing the value of *L/f* in the bridge axial direction and the bridge axial perpendicular direction is that in the bridge axial direction, the condition of *L/f* derived from single-shot shooting does not apply at the stage of image fusion. On the other hand, in the bridge axial perpendicular direction, since image fusion is not performed, there is no problem using *L/f* from Table 7. When L*/f* = 1.808 is multiplied by the image size *w* = 12533px, *W* becomes 22.66m, resulting in a deviation from the actual block length. This is presumed to be due to the influence of the accuracy of image fusion by optical flow and the fact that there were few feature points in the underside of the stiffening girder, which was the target this time. If more deformations such as other structures and corrosion can be observed other than the underside of the stiffening girder, it is considered that feature extraction will be easier. While the lack of deformation is a challenge from the perspective of ensuring the accuracy of image fusion, from the perspective of the quality of the structure, it can be said that it maintains very good accuracy.

Through image fusion by optical flow, it has become possible to identify from the images which joints are experiencing corrosion. It has become generally possible to specify areas such as near the joints or near the center of the blocks.

### 3.4 Reflection in BIM

In this section, we will describe how to reflect the corrosion area and the corrosion centroid obtained in chapter 3.3.2 into BIM, and the role of BIM.

**Fig. 22:**
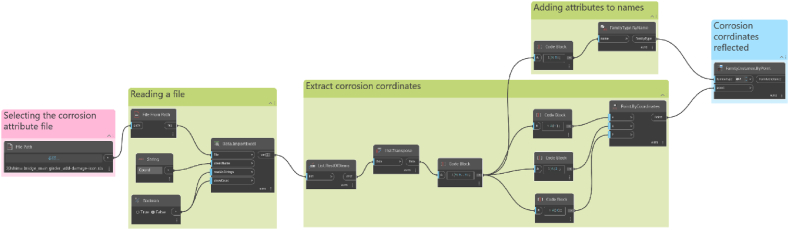
Linking IFC and corrosion data with Dynamo.

We convert to the BIM coordinate system using Eq. (14). Table 9 shows the values to be substituted into Eq. (14), and Table 10 shows the corrosion position converted to the BIM coordinate system. The names and coordinates of the corrosion are managed in a spreadsheet, and the BIM is converted into the IFC format. The corrosion icon is prepared in advance in BIM, and then, using Dynamo from Autodesk, the IFC data and coordinate data are reflected in the BIM at the corrosion parts, as shown in Fig. 22.

**Table 9:** Value to be substituted into formula (14).

θ	180°
$a_{x}$	73841 mm
$a_{y}$	2850 mm
X	-x +73841
Y	y + 2850

**Table 10:** Coordinates of corrosion location.

No.	x(mm)	y(mm)	X(mm)	Y(mm)
1	105	1002	73736	3852
2	435	1168	73406	4018
3	7036	1399	66805	4249
4	10473	946	63368	3796
5	13363	721	60478	3571
6	20640	1018	53201	3868
7	22871	240	50970	3090
8	23403	289	50438	3139
9	23757	1188	50084	4038
10	23558	1973	50283	4823

**Fig. 23:**
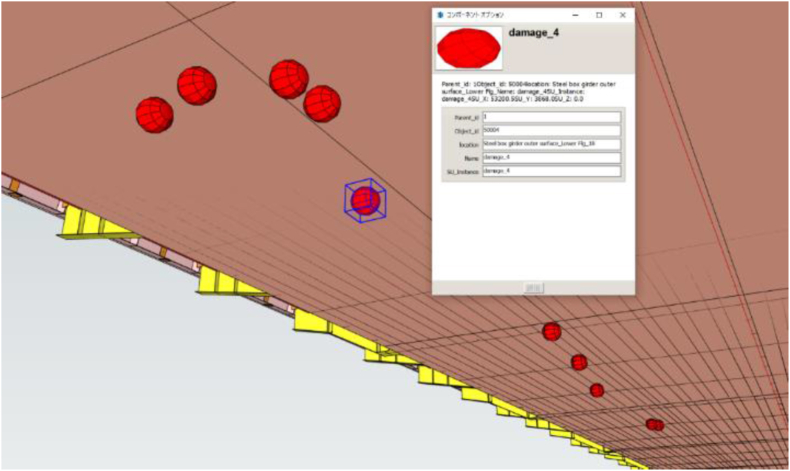
Assign corrosion location to BIM.

Only the coordinates and damage information shown in Table 10 are provided to BIM, which are then associated with the structural divisions and member names included in BIM. An example of BIM is shown in Fig. 23. BIM provides an icon for the corrosion position, from which information such as structural division, member name, and damage can be obtained in table format. As BIM allows for the management of multiple damage positions in a single data set, it contributes to the calculation of repair quantities, the acceleration of repair planning and centralization of inspection and repair history management.

In the future, by expanding the functions of BIM, providing location information filtering and damage-specific search functions, and providing display functions according to each inspection and repair, further efficiency can be expected. Also, by importing BIM into a tablet, it is possible to conduct inspections while displaying BIM on site, check the damage history, and make comparisons on the spot.

### 3.5 Applicability of this method

In this section, we discuss the extent to which the proposed inspection method can be applied to other bridges and other parts.

In the shooting of the underside of the stiffening girder of the Oshima Bridge, it was confirmed that the method proposed this time is effective. This technology targets the exterior of the stiffening girders of long-span bridge equipped with inspection vehicles. A camera needs to be installed on the inspection vehicle and directed towards the shooting surface. In the WEB of the stiffening girder of the Oshima Bridge, the camera can be fixed on a tripod and directed frontally towards the WEB surface. Therefore, shooting is possible regardless of the angle of the WEB. Furthermore, for the upper chord member, lower chord member, and diagonal member of the truss arranged parallel to the direction of movement of the inspection vehicle, this method is considered to be effective. In Japan, there are many long-span bridges such as the Akashi Kaikyo Bridge and the Seto Ohashi Bridge, and there are also bridges like the Golden Gate Bridge outside of Japan.

Additionally, inspections are being conducted on cable structures using automated robots equipped with cameras [[23], [24], [25]]. Since the distance to the subject is constant, this method is considered to be effective.

As mentioned above, there is a possibility to apply this method to all long-span bridges. However, for members perpendicular to the direction of movement of the inspection vehicle (such as cross beams, brackets, and sway bracings), it may be necessary to devise ways to identify the members from the images, such as assigning management numbers to the members. Furthermore, in complex structures, blind spots can occur due to the shooting, and thus further consideration is needed for image acquisition methods, especially in truss structures. However, this is not particularly difficult, and it can be said that this method can be applied.

## 4 Conclusion

In this study, we proposed a new method of obtaining images of the outer surface of girders using an inspection vehicle, and verified its effectiveness through experiments on actual bridges. By constructing a Mask R–CNN model integrated with PointRend and performing data augmentation using CutMix, we were able to achieve more accurate detection than traditional deep learning-based semantic segmentation. Furthermore, by performing contour tracking and dilation processing, we were able to group in necessary units and calculate the area and location of corrosion. In addition, through collaboration with BIM, in addition to the superiority that can be visually confirmed, we were able to link with structural divisions and member names, and centralize damage information. This provides important information for administrators for appropriate maintenance and decision-making, not only serving as a basis for judgment on what measures to take, but also contributing to the acceleration of repair planning.

As further advancements and efficiencies in the future, labor-saving and automation of inspection report creation can be mentioned. This includes determining the position and size of acquired images, as well as supporting the identification of damage causes and diagnosis. As a solution, it is necessary to continue cooperation with BIM and the research using VQA (Visual Question Answering) that the authors are conducting [[26], [27], [28], [29], [30]].

In this instance, we only took pictures at representative points using a camera mounted on an inspection vehicle. However, in the future, it will be necessary to install multiple cameras in the direction perpendicular to the bridge axis and conduct simultaneous shooting, to map the entire underside of the stiffening girder. Furthermore, we will extend the application to the WEB of stiffening girder, the backside of the steel floor slabs, truss members, cables, etc., to further validate the effectiveness of this method and work towards streamlining the inspection work.

## CRediT authorship contribution statement

**Kotaro Hattori:** Conceptualization, Data curation, Formal analysis, Investigation, Methodology, Project administration, Writing – original draft. **Keiichi Oki:** Formal analysis, Investigation, Methodology, Writing – original draft. **Aya Sugita:** Methodology, Software, Visualization. **Takeshi Sugiyama:** Conceptualization, Investigation, Project administration, Resources, Validation. **Pang-jo Chun:** Conceptualization, Investigation, Project administration, Supervision, Validation, Writing – review & editing.

## Declaration of competing interest

The authors declare that they have no known competing financial interests or personal relationships that could have appeared to influence the work reported in this paper.
